# Factors Associated with Cesarean Delivery Due to Intrapartum Fetal Compromise in Late-Onset Fetal Growth Restriction: A Retrospective Cohort Study

**DOI:** 10.3390/jcm15114298

**Published:** 2026-06-02

**Authors:** Dinçer Sümer, Ahmet Arif Filiz, Özgür Volkan Akbulut, Kubilay Çanga, Büşra Seçilir, Hilal Dönmez, Gülten Çirkin Tekeş, Kadriye Yakut Yücel

**Affiliations:** 1 Department of Perinatology, University of Health Sciences, Etlik City Hospital, Varlık Mahallesi Halil Sezai Erkut Cd. No:5, Yenimahalle, 06170 Ankara, Türkiye; ahmetarif_filiz@hotmail.com (A.A.F.); akbulutvolkan@yahoo.com (Ö.V.A.); kubilaycanga@hotmail.com (K.Ç.); gultencir@hotmail.com (G.Ç.T.); yakutkadriye@hotmail.com (K.Y.Y.); 2 Department of Obstetrics and Gynecology, University of Health Sciences, Etlik City Hospital, Varlık Mahallesi Halil Sezai Erkut Cd. No:5, Yenimahalle, 06170 Ankara, Türkiye; busracelik81@gmail.com (B.S.); hilaldonmez98@gmail.com (H.D.)

**Keywords:** fetal growth restriction, induction of labor, intrapartum fetal distress, cesarean delivery, systemic inflammatory indices

## Abstract

**Objective:** To investigate clinical, ultrasonographic, and hematological factors associated with cesarean delivery due to intrapartum fetal compromise in pregnancies complicated by isolated late-onset fetal growth restriction (FGR) undergoing induction of labor at 37 weeks of gestation. **Methods:** This retrospective cohort study included singleton pregnancies with isolated late-onset FGR undergoing elective induction of labor between 37 + 0 and 37 + 6 weeks of gestation. Patients who underwent cesarean delivery due to intrapartum fetal compromise constituted the study group (n = 44), whereas those who achieved vaginal delivery formed the control group (n = 100). Maternal demographic characteristics, fetal ultrasonographic findings, and systemic inflammatory indices were evaluated. Multivariable logistic regression and receiver operating characteristic (ROC) curve analyses were performed. **Results:** Gravidity, parity, induction-to-birth interval, cervical dilatation at admission, femur length, and platelet count differed significantly between the groups. In multivariable logistic regression analysis, parity remained the only independent predictor of cesarean delivery due to intrapartum fetal compromise (adjusted OR 0.421, 95% CI 0.191–0.926, *p* = 0.031). Systemic inflammatory indices and most fetal ultrasonographic parameters did not demonstrate independent predictive value. The combined multivariable model demonstrated acceptable discriminative performance (AUC 0.731, 95% CI 0.636–0.827, *p* < 0.001). Neonatal outcomes were comparable between the groups. **Conclusions:** In pregnancies complicated by isolated late-onset FGR undergoing induction of labor at 37 weeks, parity was the only independent predictor of cesarean delivery due to intrapartum fetal compromise. Routine clinical, ultrasonographic, and hematological inflammatory markers demonstrated limited independent predictive value. These findings suggest that intrapartum fetal compromise in FGR pregnancies may primarily reflect reduced fetoplacental reserve rather than isolated antenatal parameters.

## 1. Introduction

Fetal growth restriction (FGR) is commonly defined as an estimated fetal weight below the 3rd percentile for gestational age [1]. It most often occurs during the third trimester and is associated with increased risks of adverse perinatal outcomes, including fetal morbidity and mortality. In pregnancies with isolated late-onset FGR, without amniotic fluid abnormalities or abnormal umbilical artery Doppler findings, current clinical guidelines generally recommend delivery at 37 weeks of gestation to reduce the risk of intrauterine fetal demise [1,2,3]. Although vaginal delivery is generally the preferred mode of delivery in this population, a substantial proportion of fetuses develop signs of intrapartum fetal compromise during labor, which may require emergency cesarean delivery. The risk of intrapartum compromise may further increase if additional indicators of placental insufficiency, such as oligohydramnios or abnormal umbilical artery Doppler findings, are present [2,3].

Recently, systemic inflammatory indices derived from routine complete blood counts, such as neutrophil-lymphocyte ratio (NLR), systemic immune response index (SIRI), monocyte–lymphocyte ratio (MLR), pan-immune inflammation value (PIV), and systemic immune inflammation index (SII), have emerged as accessible and cost-effective markers of systemic inflammatory activation. These composite indices reflect the balance between innate immune activation (neutrophils, monocytes, and platelets) and adaptive immune regulation (lymphocytes). Previous studies have shown associations between these inflammatory markers and adverse obstetric outcomes, including preeclampsia, gestational diabetes mellitus, recurrent pregnancy loss, and late-onset FGR [4,5,6,7]. However, the predictive value of routinely available clinical, ultrasonographic, and laboratory parameters for intrapartum fetal compromise in pregnancies complicated by FGR remains unclear.

Identifying simple and readily available predictors of intrapartum fetal compromise may improve antenatal counseling, risk stratification, and delivery management in this high-risk population. Therefore, in this study, we aimed to investigate the association and discriminative performance of maternal clinical characteristics, fetal ultrasonographic findings, and hematological systemic inflammatory indices for cesarean delivery due to intrapartum fetal compromise in pregnancies complicated by isolated late-onset FGR undergoing induction of labor at 37 weeks of gestation.

## 2. Materials and Methods

### 2.1. Study Design

This retrospective cohort study was conducted at the Perinatology Clinic of Etlik City Hospital, Ankara, Türkiye. Between January 2024 and December 2025, there were 25,741 deliveries at the hospital, of which 8851 were managed in the Perinatology Clinic. During the study period, 1520 pregnancies (17.2%) complicated by fetal growth restriction were screened for eligibility.

Singleton pregnancies with cephalic presentation, reassuring fetal heart rate monitoring at admission, and planned induction of labor between 37 + 0 and 37 + 6 weeks of gestation were included. Labor induction was performed using either intravenous oxytocin infusion or a dinoprostone vaginal insert. Patients who developed intrapartum fetal compromise requiring cesarean delivery constituted the study group (n = 44), while those who achieved vaginal delivery formed the control group (n = 100).

The study was conducted in accordance with the principles of the Declaration of Helsinki. Data were obtained retrospectively from electronic medical records, and informed consent was waived by the ethics committee due to the retrospective design. Ethical approval was obtained from the Etlik City Hospital Ethics Committee (Approval No: AEŞH-BADEK2-2026-069; Date: 27 January 2026).

Due to the retrospective design, an a priori sample size calculation was not performed. A post hoc power analysis was conducted using G*Power version 3.1.9.7. Based on the observed difference in parity between the groups, the study achieved approximately 78% power to detect a moderate effect size at a two-sided alpha level of 0.05.

### 2.2. Patient Selection and Definition of Terms

Upon admission to the delivery room, all patients underwent a digital vaginal examination and ultrasonographic evaluation. Ultrasonographic assessments were performed by experienced perinatology staff using a Voluson S10 system (General Electric, Boston, MA, USA) to confirm gestational age, assess fetal viability, and evaluate fetal biometric and Doppler parameters. Gestational age was primarily determined by crown–rump length measurement obtained during the first-trimester ultrasound examination.

Fetal biometric parameters, including biparietal diameter (BPD), head circumference (HC), abdominal circumference (AC), femur length (FL), and estimated fetal weight (EFW), were measured according to the practical guidelines of the International Society of Ultrasound in Obstetrics and Gynecology (ISUOG) [8,9]. Estimated fetal weight was calculated using the Hadlock formula [10]. ΔEFW was defined as the difference between the expected mean fetal weight at 37 weeks of gestation (3028 g) and the ultrasound-estimated fetal weight measured at the time of evaluation [11]. Amniotic fluid volume was assessed using the maximum vertical pocket method, following ISUOG recommendations [8]. Umbilical artery Doppler measurements were obtained using pulsed-wave Doppler in the absence of fetal breathing and body movements. Doppler waveforms were recorded from a free-floating loop of the umbilical cord, and the pulsatility index was calculated using the autotrace function based on at least three consecutive uniform waveforms [9]. FGR was defined as an estimated fetal weight or abdominal circumference below the 3rd percentile for gestational age according to the Hadlock fetal growth reference charts, consistent with current international recommendations [1,2,3,10,11].

Cervical dilatation and effacement were assessed by digital examination, and the Bishop score was calculated for all patients. For those with a Bishop score less than 6, cervical ripening was initiated using a dinoprostone vaginal insert (Propess^®^), while intravenous oxytocin infusion was used for labor induction in patients with a Bishop score of 6 or higher. Continuous fetal heart rate monitoring with cardiotocography (CTG) was performed throughout labor. Nonreassuring fetal status was defined according to the FIGO consensus guideline as abnormal fetal heart rate monitoring patterns, including recurrent late decelerations, prolonged decelerations, persistent fetal bradycardia, fetal tachycardia, reduced baseline variability, and recurrent variable decelerations suggestive of fetal hypoxia [12]. Decisions regarding cesarean delivery due to intrapartum fetal compromise were made by experienced perinatology specialists based on continuous CTG monitoring, overall intrapartum clinical assessment, and institutional management protocols derived from FIGO recommendations.

Immediately after delivery and before umbilical cord clamping, the cord was clamped on both the fetal and placental sides. Approximately 2 mL of umbilical arterial blood was collected from the isolated cord segment using a syringe, and cord blood pH analysis was performed immediately.

**Figure 1:**
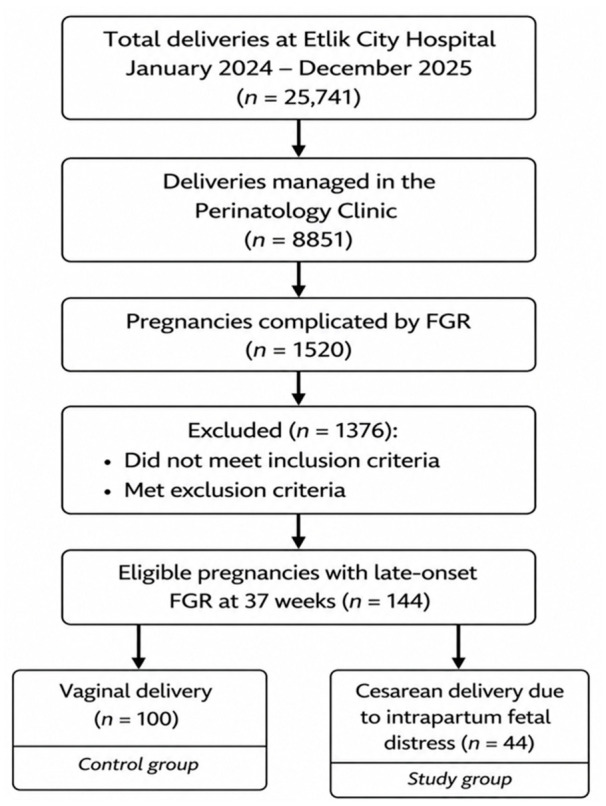
Flowchart of patient selection and study design.

Patients were excluded if they met any of the following criteria: multiple pregnancy; previous uterine scar; contraindications to vaginal delivery or labor induction, including breech presentation, transverse lie, placenta previa, vasa previa, previous classical hysterotomy, or nonreassuring CTG at admission; chronic systemic disease; active labor at admission; gestational age outside the specified range; planned primary cesarean delivery for any indication; fetal chromosomal or structural anomalies; pregnancies complicated by gestational diabetes mellitus, hypertensive disorders of pregnancy, intrahepatic cholestasis, or other major obstetric comorbidities; systemic steroid usage; known drug allergy; amniotic fluid abnormalities including oligohydramnios, polyhydramnios, or preterm premature rupture of membranes; abnormal umbilical artery Doppler findings such as absent or reversed end-diastolic flow or elevated Doppler indices; single umbilical artery; cesarean delivery performed due to failed induction or arrest of labor rather than intrapartum fetal compromise; and patients whose antenatal follow-up was not performed at our institution. The flowchart of the study population is presented in Figure 1.

### 2.3. Data Collection and Laboratory Parameters

Clinical and laboratory data were collected retrospectively from the hospital electronic medical record system. Hematological and systemic inflammatory indices, including neutrophil–lymphocyte ratio (NLR), systemic immune response index (SIRI), monocyte–lymphocyte ratio (MLR), pan-immune inflammation value (PIV), systemic immune inflammation index (SII), and platelet–lymphocyte ratio (PLR), were calculated using routine complete blood count parameters. The indices were defined as follows: NLR, neutrophil count divided by lymphocyte count; MLR, monocyte count divided by lymphocyte count; SIRI, neutrophil count multiplied by monocyte count divided by lymphocyte count; PIV, neutrophil count multiplied by platelet count multiplied by monocyte count divided by lymphocyte count; SII, platelet count multiplied by neutrophil count divided by lymphocyte count; and PLR, platelet count divided by lymphocyte count [5,6,7]. These inflammatory indices were selected based on previous studies evaluating systemic inflammatory activation in placental dysfunction and late-onset fetal growth restriction.

### 2.4. Statistical Analysis

Statistical analyses were conducted using the Statistical Package for the Social Sciences Version 22.0 (IBM Corporation, Armonk, New York, NY, USA). The distribution of variables was assessed with the Kolmogorov–Smirnov test. As the variables did not follow a normal distribution, continuous variables were reported as median (interquartile range, IQR) and compared between groups using the Mann–Whitney U test. Categorical variables were presented as number and percentage (n, %) and analyzed using the chi-square test or Fisher’s exact test.

Variables showing statistical significance in univariate analysis (*p* < 0.05), along with clinically relevant parameters, were included in a multivariable logistic regression model to identify independent predictors of cesarean delivery due to intrapartum fetal compromise. The strength of associations was expressed as odds ratios (ORs) and adjusted odds ratios (aORs) with corresponding 95% confidence intervals (CIs). Multicollinearity among independent variables was assessed using variance inflation factor (VIF) and tolerance statistics. Due to conceptual overlap and potential multicollinearity between gravidity and parity, only parity was included in the final multivariable model.

Receiver operating characteristic (ROC) curve analysis was used to evaluate the discriminative performance of individual predictors and the combined multivariable model. Calibration was assessed using the Hosmer–Lemeshow goodness-of-fit test. A *p* value < 0.05 was considered statistically significant.

## 3. Results

Maternal age, history of abortion, use of assisted reproductive technology, smoking status, first-trimester PAPP-A MoM and hCG MoM values, body mass index (BMI) at the first trimester and at delivery, and gestational weight gain were comparable between the groups (all *p* > 0.05). However, gravidity (*p* = 0.035) and parity (*p* = 0.005) were significantly higher in the vaginal delivery group.

For intrapartum characteristics, the induction-to-birth interval was significantly longer in the vaginal delivery group compared with the cesarean delivery group (11.5 [11.0] vs. 6.5 [13.0] h, *p* = 0.039). Cervical dilatation at admission was also significantly greater in the vaginal delivery group (1.0 [2.0] cm vs. 0.0 [1.0] cm, *p* = 0.023). Cervical effacement, induction method, and betamethasone administration did not differ significantly between the groups.

Among fetal biometric parameters, femur length was significantly greater in the vaginal delivery group (*p* = 0.018), while biparietal diameter, head circumference, abdominal circumference, maximum vertical pocket, estimated fetal weight, and ΔEFW were comparable between the groups (all *p* > 0.05) (Table 1).

Laboratory parameters, including hemoglobin level, leukocyte count, neutrophil count, lymphocyte count, monocyte count, systemic inflammatory indices (SIRI, SII, MLR, NLR, and PIV), fibrinogen level, and albumin level, were comparable between the groups. However, platelet count was significantly higher in the cesarean delivery group (*p* = 0.027) (Table 2).

To minimize the potential confounding effect of antenatal corticosteroid exposure on hematological and inflammatory parameters, an additional sensitivity analysis was performed after excluding patients who had received betamethasone within the previous two weeks. The overall findings remained unchanged, and inflammatory indices, including NLR, SII, SIRI, MLR, and PIV, continued to show no significant association with cesarean delivery due to intrapartum fetal compromise.

Regarding neonatal outcomes, birth weight was significantly lower in the cesarean delivery group compared with the vaginal delivery group (2300 [315] g vs. 2400 [200] g, *p* = 0.018). Neonatal sex distribution, 1 min and 5 min Apgar scores, NICU admission rates, cord blood pH values, and base excess levels were similar between the groups (Table 3).

In the univariate logistic regression analysis, parity (OR = 0.430, 95% CI: 0.223–0.807, *p* = 0.009), cervical dilatation (OR = 0.641, 95% CI: 0.448–0.917, *p* = 0.015), and platelet count (OR = 1.006, 95% CI: 1.001–1.012, *p* = 0.025) were significantly associated with cesarean delivery due to intrapartum fetal compromise. These variables were then included in the multivariable logistic regression model. In the multivariable analysis, parity remained the only independently associated factor of cesarean delivery (adjusted OR = 0.421, 95% CI: 0.191–0.926, *p* = 0.031). However, cervical dilatation (aOR = 0.764, 95% CI: 0.543–1.076, *p* = 0.123) and platelet count (aOR = 1.005, 95% CI: 0.999–1.012, *p* = 0.084) were not independently associated with cesarean delivery after adjustment for potential confounders (Table 4). Multicollinearity among independent variables was assessed using the variance inflation factor (VIF) and tolerance statistics, and no significant multicollinearity was found in the final multivariable model.

The multivariable model explained approximately 18.9% of the variance in cesarean delivery due to intrapartum fetal compromise, as indicated by the Nagelkerke R^2^ value. The overall logistic regression model was statistically significant based on the omnibus test of model coefficients (χ^2^ = 17.450, df = 3, *p* = 0.001). Calibration analysis using the Hosmer–Lemeshow goodness-of-fit test showed acceptable agreement between predicted and observed outcomes (χ^2^ = 13.070, df = 8, *p* = 0.109).

Receiver operating characteristic (ROC) curve analysis showed modest but statistically significant discriminative performance for parity in predicting cesarean delivery due to intrapartum fetal compromise (AUC = 0.630, 95% CI: 0.535–0.726, *p* = 0.012). Cervical dilatation showed similar discriminative ability (AUC = 0.622, 95% CI: 0.520–0.725, *p* = 0.018).

**Figure 2:**
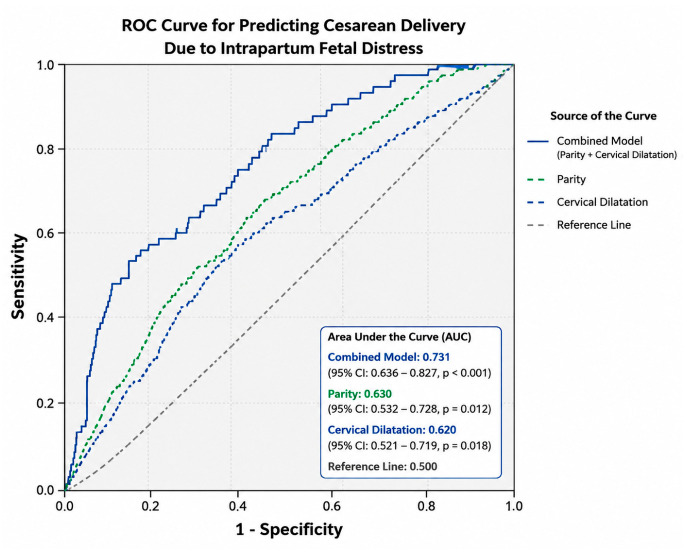
Receiver operating characteristic (ROC) curves for parity, cervical dilatation, and the combined multivariable model in predicting cesarean delivery due to intrapartum fetal compromise in isolated late-onset fetal growth restriction. The combined model demonstrated improved discriminative performance compared with the individual predictors alone (AUC: 0.731, 95% CI: 0.636–0.827, *p* < 0.001).

To further assess the predictive performance of the multivariable model, ROC curve analysis was performed using the predicted probabilities from the combined logistic regression model. The combined model showed acceptable discriminative ability for predicting cesarean delivery due to intrapartum fetal compromise, with an AUC of 0.731 (95% CI: 0.636–0.827, *p* < 0.001), indicating improved performance compared with the individual predictors alone (Figure 2).

## 4. Discussion

In the present study, we evaluated clinical and laboratory predictors of cesarean delivery due to intrapartum fetal compromise in pregnancies with isolated fetal growth restriction undergoing elective induction of labor at 37 weeks of gestation. The main finding was that parity remained independently associated with the risk of cesarean delivery after multivariable adjustment. Additionally, cervical dilatation at admission and platelet count were associated with cesarean delivery in univariate analysis, but these associations did not persist after adjustment for potential confounders. Overall, these findings suggest that maternal obstetric characteristics, particularly parity, may play a more significant role in determining intrapartum outcomes than isolated laboratory-derived parameters in pregnancies complicated by fetal growth restriction.

Previous studies have sought to identify predictors of cesarean delivery in pregnancies complicated by fetal growth restriction undergoing induction of labor. In a retrospective study of 146 women induced after 36 weeks of gestation with an unfavorable cervix, maternal age over 39 years, nulliparity, and abnormal umbilical artery Doppler velocimetry were identified as independent predictors of cesarean delivery [13]. Notably, neonatal outcomes did not differ significantly between women who delivered vaginally and those who had a cesarean delivery, suggesting that induction of labor may remain a reasonable management strategy even with an unfavorable cervix.

In our study, 44 of the 144 women (30.6%) required cesarean delivery due to intrapartum fetal compromise, while the remaining patients had vaginal deliveries. Consistent with the previous study, neonatal outcomes were comparable between the groups. However, unlike previous findings, none of the evaluated maternal demographic characteristics, laboratory-derived inflammatory indices, or fetal ultrasonographic parameters showed independent predictive value after multivariable adjustment, except for parity, which remained the only independent predictor in our analysis. This observation may reflect the multifactorial nature of intrapartum fetal compromise in pregnancies complicated by fetal growth restriction, where reduced fetoplacental reserve and fetal tolerance to labor may be influenced by complex physiological mechanisms rather than by isolated antenatal parameters.

In addition, birth weight was significantly lower in the cesarean delivery group. Although birth weight did not remain independently associated with cesarean delivery in regression analyses, this finding may still reflect a greater degree of placental insufficiency and reduced fetoplacental reserve in fetuses developing intrapartum compromise. It is also possible that the observed association between parity and delivery outcome may partly reflect differences in fetal vulnerability or severity of growth restriction that were not fully captured by routine antenatal parameters.

Another study highlighted the importance of maternal and cervical characteristics in predicting the success of labor induction in pregnancies complicated by fetal growth restriction. In a retrospective cohort of 161 pregnancies induced with a dinoprostone vaginal insert, 117 (73%) achieved successful vaginal delivery, while 44 (27%) did not achieve adequate cervical ripening [14]. Parity and Bishop score were identified as significant predictors of successful vaginal birth, and the predictive model demonstrated good discriminative performance.

Similarly, in our cohort, 100 of 144 women (69.4%) achieved vaginal delivery, while 44 (30.6%) required cesarean delivery due to intrapartum fetal compromise after induction with either a dinoprostone vaginal insert or oxytocin infusion. Consistent with the previous study, parity remained independently associated with delivery outcome in our multivariable analysis, supporting the idea that maternal obstetric history may play an important role in determining intrapartum outcomes in pregnancies complicated by fetal growth restriction undergoing induction of labor.

One objective of this study was to evaluate whether systemic inflammatory indices derived from routine complete blood count parameters could predict intrapartum fetal compromise in pregnancies complicated by fetal growth restriction. However, none of the evaluated indices, including NLR, SII, SIRI, MLR, and PIV, differed significantly between the groups or demonstrated independent predictive value for cesarean delivery.

Previous studies have suggested that systemic inflammatory markers may be associated with placental dysfunction and the development of late-onset fetal growth restriction [4,5]. However, their role in predicting intrapartum fetal compromise remains uncertain. Intrapartum fetal compromise is likely determined by the dynamic balance between fetal oxygen demand and fetoplacental reserve during labor rather than by baseline systemic inflammatory status alone, as fetuses with placental insufficiency may have limited tolerance to hypoxic stress during uterine contractions [2,15,16,17]. Our findings therefore suggest that although these hematological markers may reflect underlying placental pathology, they may not be sufficiently sensitive to predict acute intrapartum fetal compromise in otherwise stable pregnancies undergoing induction of labor.

Another possible explanation for the lack of association between systemic inflammatory indices and intrapartum fetal compromise may be related to the physiological inflammatory processes that occur during labor. Human parturition is widely recognized as a sterile inflammatory process characterized by activation of maternal immune pathways, leukocyte infiltration into reproductive tissues, and increased production of inflammatory mediators such as cytokines and prostaglandins [18,19]. Because these inflammatory responses occur physiologically during normal labor, hematological inflammatory markers measured before or during labor may partly reflect normal immunological adaptation rather than pathological inflammatory activation associated with fetal compromise.

Intrapartum complications remain a significant contributor to adverse perinatal outcomes, including hypoxic–ischemic injury and stillbirth. In many cases, fetal hypoxia may develop gradually due to reduced fetoplacental reserve before labor begins. Previous studies have shown that antenatal markers of placental dysfunction, such as the cerebroplacental ratio (CPR) and maternal placental growth factor (PlGF) levels, can help identify fetuses at increased risk of intrapartum compromise. For example, the PROMISE study proposed a screening strategy that combines CPR and maternal PlGF levels to predict intrapartum fetal compromise and guide clinical decision-making at term [20]. These findings highlight the importance of identifying pregnancies with limited fetoplacental reserve before labor, especially in cases of fetal growth restriction, where the risk of intrapartum intolerance is higher. In the present study, we evaluated maternal demographic and clinical characteristics, systemic inflammatory indices, and fetal ultrasonographic parameters as potential predictors of intrapartum fetal compromise. However, none of these parameters, except parity, showed independent predictive value for cesarean delivery in our cohort.

The main strength of this study is its relatively homogeneous population, consisting of pregnancies with isolated late-onset fetal growth restriction without major obstetric comorbidities and undergoing elective induction of labor at 37 weeks of gestation. This design allowed for a focused evaluation of clinical, ultrasonographic, and hematological factors associated with intrapartum fetal compromise in a well-defined cohort. However, the highly selective nature of the study population may limit the generalizability of the findings and increase the potential for selection bias. Nevertheless, the restrictive inclusion and exclusion criteria were intentionally applied to achieve a more homogeneous cohort and to minimize potential confounding factors that could influence intrapartum outcomes in pregnancies complicated by fetal growth restriction.

However, several limitations should be acknowledged. First, the retrospective design may have introduced selection bias and residual confounding. Nevertheless, because CTG interpretation may involve some degree of interobserver variability and clinical judgment, the possibility of observer and indication bias cannot be completely excluded. Second, the sample size and number of cesarean delivery events were relatively modest. Although post hoc power analysis demonstrated acceptable power for detecting moderate effect sizes, the study may have been underpowered to detect small or modest associations, particularly for the evaluated systemic inflammatory indices. Therefore, the possibility of type II error cannot be excluded. In addition, the limited number of cesarean delivery events may have affected model stability and restricted the feasibility of more advanced internal validation techniques. Third, cerebroplacental ratio (CPR), which is currently considered one of the most validated antenatal Doppler markers for predicting intrapartum compromise in late-onset fetal growth restriction, was not systematically available because of the retrospective design and the non-standardized nature of Doppler assessments in routine clinical practice during the study period. The absence of CPR data may have limited the interpretation of the negative ultrasonographic findings, as CPR could potentially provide additional information regarding fetoplacental reserve and fetal tolerance to labor. Finally, the study was conducted at a single tertiary referral center, which may limit the generalizability of the findings.

## 5. Conclusions

In conclusion, among pregnancies with fetal growth restriction undergoing induction of labor at 37 weeks of gestation, parity was the only independent associated factor of cesarean delivery due to intrapartum fetal compromise. Maternal demographic characteristics, systemic inflammatory indices, and fetal ultrasonographic parameters did not show independent predictive value. These findings suggest that intrapartum fetal compromise in pregnancies with fetal growth restriction may primarily reflect reduced fetoplacental reserve rather than isolated antenatal parameters. Further prospective studies with larger cohorts are needed to better identify reliable predictors of intrapartum fetal intolerance in this population.

## Figures and Tables

**Table 1:** Comparison of maternal demographic, obstetric, intrapartum, and fetal ultrasonographic characteristics between the vaginal delivery and cesarean delivery groups.

Parameter	Vaginal Delivery (n = 100)	Cesarean Delivery Due to İntrapartum Fetal Compromise (n = 44)	*p* Value
**Maternal characteristics**			
Age (years)	26.0 (7.0)	25.0 (6.0)	0.185
Gravidity (n)	2.0 (2.0)	1.0 (1.0)	*0.035*
Parity (n)	0.5 (1.0)	0.0 (1.0)	*0.005*
Abortus (n)	0.0 (0.0)	0.0 (0.0)	0.875
Assisted reproductive technology	1 (1%)	0 (0%)	1.000
Smoking	7 (7.0%)	6 (13.0%)	0.073
PAPP-A MoM at first trimester	0.83 (1.0)	1.09 (1.0)	0.302
HCG MoM at first trimester	0.87 (1.0)	0.82 (1.0)	0.356
BMI at first trimester (kg/m^2^)	22.0 (6.0)	23.0 (5.0)	0.318
Weight gain during pregnancy (kg)	10.0 (7.0)	10.0 (5.0)	0.331
BMI at delivery (kg/m^2^)	26.0 (7.0)	28.0 (5.0)	0.065
**Intrapartum characteristics**			
Induction to birth interval (hours)	11.5 (11.0)	6.5 (13.0)	*0.039*
Cervical dilatation (cm)	1.0 (2.0)	0.0 (1.0)	*0.023*
Cervical effacement	0.0 (0.0%)	0.0 (0.0%)	0.120
Induction method Dinoprostone vaginal insert Intravenous Oxytocin infusion	68 (68.0%) 32 (32.0%)	31 (70.5%) 13 (29.5%)	0.770
Betamethasone administration within the previous two weeks	7 (7.8%)	6 (13.6%)	0.282
**Fetal ultrasound parameters**			
Biparietal Diameter (mm)	86.0 (5.0)	87.0 (5.0)	0.873
Head Circumference (mm)	315.0 (14.0)	311.0 (13.0)	0.609
Abdominal Circumference (mm)	294.5 (15.0)	295.0 (10.8)	0.761
Femur Length (mm)	67.0 (4.0)	66.0 (3.8)	*0.018*
Maximum Vertical Pocket (MVP) (mm)	44.0 (14.0)	48.0 (20.0)	0.577
Estimated Fetal Weight (EFW) (g)	2390 (300)	2357 (248)	0.676
∆EFW (g)	−656 (300)	−672 (248)	0.282

IQR: interquartile range, BMI: Body mass index, PAPP-A pregnancy-associated Plasma Protein-A, Data are presented as median (interquartile range, IQR) or number (percentage). Continuous variables were compared using the Mann–Whitney U test and categorical variables using the chi-square or Fisher’s exact test. Statistically significant values are presented in *italics*.

**Table 2:** Comparison of hematological and systemic inflammatory markers between the study groups.

Parameter	Vaginal Delivery (n = 100)	Cesarean Delivery Due to İntrapartum Fetal Compromise (n = 44)	*p* Value
Hemoglobin level (g/dL)	12.0 (1.8)	11.9 (1.1)	0.881
Leukocyte count (×10^3^/µL)	10.0 (3.0)	10.0 (2.8)	0.906
Neutrophil count (×10^3^/µL)	7.9 (2.8)	7.9 (2.6)	0.739
Lymphocyte count (×10^3^/µL)	1.8 (0.7)	1.9 (0.6)	0.893
Monocyte count (×10^3^/µL)	0.6 (0.3)	0.6 (0.3)	0.773
Platelet count (×10^3^/µL)	232.0 (82.5)	261.5 (84.5)	*0.027*
SIRI	2.65 (1.88)	2.38 (1.42)	0.731
SII	982 (651)	1102 (618)	0.217
MLR	0.33 (0.16)	0.33 (0.11)	0.979
NLR	4.15 (2.13)	4.06 (2.18)	0.742
PIV	573.9 (525.8)	629.4 (412.3)	0.440
Fibrinogen level (mg/dL)	474 (97)	483 (102)	0.536
Albumin level (g/L)	37.0 (3.0)	37.0 (4.0)	0.850

IQR: Interquartile range; NLR: Neutrophil–lymphocyte ratio; SIRI: Systemic immune response index; MLR: Monocyte–lymphocyte ratio; PIV: Pan-immune inflammation value; SII: Systemic immune inflammation index. Data are presented as median (IQR). Comparisons were performed using the Mann–Whitney U test. Statistically significant values are presented in *italics*.

**Table 3:** Comparison of neonatal outcomes between the vaginal delivery group and the cesarean delivery due to fetal distress group.

Parameter	Vaginal Delivery (n = 100)	Cesarean Delivery Due to İntrapartum Fetal Compromise (n = 44)	*p* Value
Birth weight (g)	2400 (200)	2300 (315)	*0.018*
Gender Female Male	59 (59.0%) 41 (41.0%)	21 (47.7%) 23 (52.3%)	0.210
1 min Apgar score	8 (1)	8 (2)	0.447
5 min Apgar score	9 (1)	9 (2)	0.293
NICU admission	3 (3.0%)	3 (6.8%)	0.370
Cord blood pH	7.30 (0.02)	7.33 (0.08)	0.120
Cord blood base excess	−4.7 (3.09)	−2.5 (5.62)	0.093

NICU: Neonatal intensive care unit. Data are presented as median (IQR) or n (%). Continuous variables were analyzed using the Mann–Whitney U test. Statistically significant values are presented in *italics*.

**Table 4:** Univariate and multivariate logistic regression analysis of factors associated with cesarean delivery due to fetal distress.

		Univariate			Multivariate	
Parameter	OR	95% CI	*p* Value	aOR	95% CI	*p* Value
Parity	0.430	0.223–0.807	*0.009*	0.421	0.191–0.926	*0.031*
Induction to delivery interval	0.974	0.929–1.021	0.277	-	-	**-**
Cervical dilatation	0.641	0.448–0.917	*0.015*	0.764	0.543–1.076	0.123
Platelet count	1.006	1.001–1.012	*0.025*	1.005	0.999–1.012	0.084
Femur Length	0.961	0.874–1.057	0.410	-	-	**-**
EFW	1.000	0.998–1.001	0.801	-	-	**-**
∆EFW	1.000	0.998–1.001	0.564	-	-	**-**

EFW: Estimated Fetal Weight Statistically significant values are presented in *italics*.

## Data Availability

The datasets used and/or analyzed during the current study are available from the corresponding author on reasonable request.

## Abbreviations

AC	Abdominal circumference
aOR	adjusted Odds Ratio
AUC	Area under the curve
BMI	Body mass index
BPD	Biparietal diameter
CPR	Cerebroplacental ratio
CTG	Cardiotocography
ΔEFW	Delta Estimated fetal weight
EFW	Estimated fetal weight
FGR	Fetal growth restriction
FIGO	International Federation of Gynecology and Obstetrics
FL	Femur length
HC	Head circumference
hCG	Human chorionic gonadotrophin
IQR	Interquartile range
ISUOG	International Society of Ultrasound in Obstetrics and Gynecology
MLR	Monocyte-lymphocyte ratio
MoM	Multiple of median
NICU	Neonatal intensive care unit
NLR	Neutrophil-lymphocyte ratio
OR	Odds ratio
PAPP-A	Pregnancy-associated Plasma Protein-A
PIV	Pan-immune inflammation value
PlGF	Placental growth factor
PLR	Platelet–lymphocyte ratio
ROC	Receiver operating characteristic
SD	Standard deviation
SII	Systemic immune inflammation index
SIRI	Systemic immune response index
VIF	Variance inflation factor
